# The Development of Simple Scoring System to Predict Urinary Tract Infection (UTI) in Patients with Stroke

**DOI:** 10.1155/2024/2512824

**Published:** 2024-05-27

**Authors:** In-Hui Pak, Se-Ryong Han, Chol-Ho Sin, Hyo-Song Kim, Un-Ryong Rim

**Affiliations:** ^1^Faculty of Biomedical Engineering, Kim Chaek University of Technology, Pyongyang, Democratic People's Republic of Korea; ^2^Neurology Department, Pyongyang Medical College Hospital, Pyongyang, Democratic People's Republic of Korea; ^3^Chongjin Medical College Hospital, Chongjin, Democratic People's Republic of Korea; ^4^Institute of Engineering, Kim Chaek University of Technology, Pyongyang, Democratic People's Republic of Korea

## Abstract

Urinary tract infection is a frequent problem after stroke. Although prior scoring systems for UTI after stroke have been developed, we developed a simple scoring system for all types of stroke in our own. The study was designed on retrospective data. The population includes 1496 patients with stroke who had been admitted at the Neurology Department of Pyongyang Medical College Hospital between January 2010 and August 2019. The patients were diagnosed with confirmed CT and MRI. Urinary tract infection (UTI) was diagnosed through urine culture: more than 100,100 colony-forming units per millimeter in patients with signs and symptoms. The UTI prediction scoring system was developed by means of the variables available on admission. The variables with significant difference between the non-UTI group and the UTI group were age (non-UTI versus UTI, 56.4 ± 7.2 vs. 59.0 ± 12.8; *p* < 0.001), female (244 (24.2) vs. 176 (36.1), *p* < 0.001), 300 ≦ SI (smoking index) (16 (2.4) vs. 48 (12.0), *p* < 0.001), alcohol > 25 g/d (292 (29.0) vs. 184 (37.7), *p* < 0.001), poststroke hyperglycemia (120 (10.3) vs. 163 (33.4), *p* < 0.001), indwelling of urinary catheter (157 (15.6) vs. 351 (72.0), *p* < 0.001), GCS (Glasgow Coma Scale) on admission (11.2 ± 3.9 vs. 8.5 ± 4.0, *p* = 0.038), and WFNS (World Federation of Neurosurgeons) (in subarachnoid hemorrhage) on admission (2.9 ± 1.7 vs. 3.5 ± 1.5, *p* < 0.001). The UTI prediction score ranged from 0 to 8 and produced an AUC (area under curve) of 0.800. The optimal cutoff point was 2.5 (sensitivity 64.3% and specificity 79.9%). So, the score ≧ 3 was the optimal score for the prediction of UTI after stroke.

## 1. Introduction

Stroke remains a tremendous public health burden with approximately 795,000 people affected yearly. Stroke is the leading cause of major long-term disability in adults and the third leading cause of death in developed countries [1, 2].

Infections can result in electrolyte disturbances, hypoxia, and elevated body temperature, which have, theoretically, a negative effect on the vitality of neurons within the ischemic penumbra [3, 4]. Fever may increase the cerebral metabolic demands, change the blood-brain barrier permeability, and promote acidosis and release of excitatory amino acids [5].

The revealed risk of hospital-acquired UTI varies significantly in the available studies with risks ranging from 3.7% to 65.8% [6].

Poststroke infections such as pneumonia, urinary tract infection (UTI), and bacteremia are problematic because they increase the risk of mortality and disability among the patients with stroke through fever, immobilization, and end-organ damage resulting from septic shock [1, 3]. Simultaneously they increase the length of the stay in the hospital and hospital costs [7, 8]. Although patient-related factors such as stroke severity and age are threatening the outcome following stroke, poststroke infections may also play a long-term role [9–12]. Early identification of risk factors for the development of such infections is an effective way to prevent patients from undesirable outcomes.

Urinary tract infection is a frequent problem after stroke, occurring in 11 to 15 percent of patients followed for up to three months ([7, 13]) and it constitutes a serious complication (i.e., prolonged, immediately life-threatening, or resulting in hospitalization or death) in about 1 percent [14]. Therefore, we conducted a retrospective study to develop a new simple prediction score for UTI in patients with stroke by using the variables available on admission.

## 2. Methods

### 2.1. Study Population and Data Collection

The study was designed on retrospective data. The population includes 1496 patients with stroke who had been admitted at the Neurology Department of Pyongyang Medical College Hospital between January 2010 and August 2019.

A retrospective analysis of collected data of all patients with UTI was performed using previously described methods [6, 15].

The patients were diagnosed with confirmed CT and MRI. UTI was diagnosed through urine culture: more than 100,100 colony-forming units per millimeter in patients with signs and symptoms including pain or a burning feeling when you urinate, the need to urinate often, the need to urinate suddenly or in a hurry, blood in the urine, fever, back pain, nausea, or vomiting [16].

And the following factors were assessed: age, gender, season, smoking, alcohol, diabetes, poststroke hyperglycemia, the state of indwelling urinary catheter, GCS (Glasgow Coma Scale), NIHSS (National Institute of Health Stroke Scale), and WFNS (World Federation of Neurosurgeons) (in subarachnoid hemorrhage: SAH) on admission. We assessed smoking by using the following smoking index (SI): SI=cigarettes per day × years of smoking.

All variables were obtained from intro-net in our hospital, and the present study was approved by the Ethics Committee of Pyongyang Medical College Hospital.

### 2.2. Statistical Analysis

All data were analyzed with SPSS 20.0 (IBM Corporation, Armonk, NY, USA). We divided the patients into two groups according to the development of UTI. We compared all the variables of interest between two groups: i.e., patients with and without UTI. The Pearson chi-square test was used for proportions. The optimal cutoff for each continuous variable to discriminate two groups was obtained by ROC (receiver operating characteristics) curves. The points for the variables in the score were set using beta coefficients from the adjusted logistic regression model for poststroke UTI. Logistic regression was then used to evaluate which prediction score cutoff was most predictive. *p* value less than 0.05 was significant.

## 3. Results

**Figure 1:**
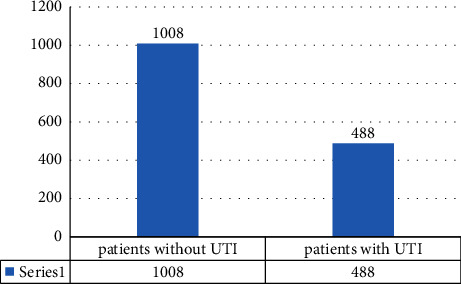
Distribution of patients with UTI after stroke.

Among 1496 patients with stroke, 488 (32.6%) patients were found to have UTI in our settings (Figure 1). The baseline characteristics of the patients are shown in Table 1.

As shown in Table 1, the variables with significant difference between the non-UTI group and the UTI group are age (non-UTI versus UTI, 56.4 ± 7.2 vs. 59.0 ± 12.8; *p* < 0.001), female (244 (24.2) vs. 176 (36.1), *p* < 0.001), 300 ≦ SI (smoking index) (16 (2.4) vs. 48 (12.0), *p* < 0.001), alcohol >25 g/d (292 (29.0) vs. 184 (37.7), *p* < 0.001), poststroke hyperglycemia (120 (10.3) vs. 163 (33.4), *p* < 0.001), indwelling of urinary catheter (157 (15.6) vs. 351 (72.0), *p* < 0.001), GCS on admission (11.2 ± 3.9 vs. 8.5 ± 4.0, *p*=0.038), and WFNS (in SAH) on admission (2.9 ± 1.7 vs. 3.5 ± 1.5, *p* < 0.001), and they are used to predict UTI after stroke. It can be seen from the ROC curve in Figure 1 that the optimal cutoff point of the continuous variables with high sensitivity and specificity including age, GCS on admission, and WFNS (in the case of SAH) is as follows: age > 57.5 (sensitivity 75.3%, specificity 87.5%, and AUC (area under the curve) 0.799, *p* < 0.001), GCS<11.5 (sensitivity 69.6%, specificity 60.3%, and AUC 0.694, *p* < 0.001), and WFNS in subarachnoid hemorrhage > 4.5 (sensitivity 71.7%, specificity 55.7%, and AUC 0.7609, *p*=0.817). WFNS was excluded from the continuous variables because there is no significance. The significant factors in the UTI group were assessed by using multivariate logistic regression analysis (see Table 2).

The point proportional to the regression coefficient of each factor was assigned to the factor as shown in Table 3, and it can be seen that the total score is 8.

**Figure 2:**
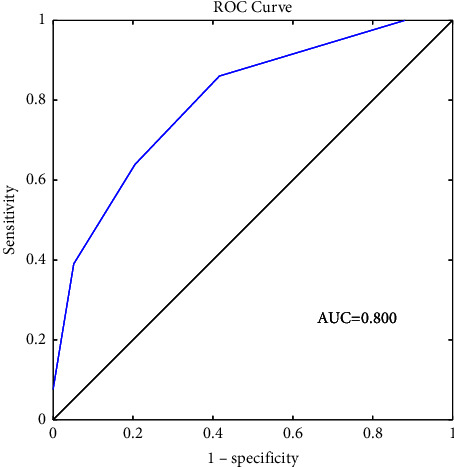
Receiver operating characteristic curve for the UTI prediction score.

With 8-point scoring system, we evaluated the optimal cutoff point by using ROC curve analysis. The UTI prediction score ranged from 0 to 8 and produced an AUC of 0.800 (Figure 2).

The optimal cutoff point was 2.5 (sensitivity 64.3% and specificity 79.9%). So score ≧ 3 was the optimal score for the prediction of UTI after stroke. If validated, the scoring system will help doctors to predict UTI after stroke and take the appropriate measures.

## 4. Discussion

Urinary tract infections, also called “UTIs,” are infections that affect either the bladder or the kidneys. Bladder infections are more common than kidney infections. Bladder infections happen when bacteria get into the urethra and travel up into the bladder. Kidney infections happen when the bacteria travel even higher up into the kidneys. Both bladder and kidney infections are more common in women than men.

The symptoms of bladder infection include pain or a burning feeling when you urinate, the need to urinate often, the need to urinate suddenly or in a hurry, and blood in the urine, and the symptoms of a kidney infection can include the symptoms of a bladder infection, but kidney infections can also cause fever, back pain, and nausea or vomiting [16].

Furthermore, urinary tract infection is a frequent problem after stroke, occurring in 11 to 15 percent of patients followed for up to three months [7, 13]. Previous studies in stroke patients have cited an extensive variety of rates for UTI, from as low as 3% up to as high as 44% [17]. Indredavik and colleagues reported that hospital incidence of UTIs is very common among stroke patients 16% in the first week and 27.9% at 3 months [18]. Also with a multicenter study of 311 consecutive acute stroke admissions, the in-hospital incidence of UTI was 24% [19]. In another previous study of 1455 patients of acute ischemic stroke patients recruited to a randomized controlled trial, the authors found an incidence of 17.2% of UTI [20]. Stott and colleagues denoted that the wide variations of in-hospital UTI rates in stroke patients are likely to be a result of differences in the selection of patients and in case mix [6].

In our study, around one in 3 patients (488/1496) was found to have UTI during poststroke through urinalysis.

We developed a simple prediction score system for UTI during poststroke hospitalization by means of the simple clinical and demographic variables available on admission including alcohol drinking, age, female, GCS on admission, poststroke hyperglycemia, smoking, and indwelling urinary catheter. This score system can be applied to all types of stroke: ischemic stroke, intracerebral hemorrhage, and subarachnoid hemorrhage. The risk factors for UTI are variable. The placement of an indwelling bladder catheter is an important risk factor for infection, and the duration of catheterization is directly related to the risk of urinary tract infection [21]. Because of this risk, the use of indwelling urinary catheters should be avoided whenever possible [22]. The use of external catheter systems (i.e., condom catheters for men and adhesive urinary pouches for women) or intermittent catheterizations are alternatives that may be associated with a lower risk of urinary tract infections compared with an indwelling urethral catheter. However, supporting data are scant [23].

Predictive scores for UTI have been developed in prior research studies [24]. They have mentioned that age ≧ 70, diabetes, and baseline NIHSS (National Institute of Health Stroke Scale) were powerful factors to predict UTI after stroke [24]. Of course, these scores are valuable to predicting stroke-associated pneumonia in their area. However, we were trying to develop our new scoring system by using the variables on admission in our region. Our study demonstrates that a score using the variables available on admission can predict the UTI after stroke (ACU = 0.800, *p* < 0.001). Our score may efficiently prove valuable in an effort to prevent UTI. Again, in the prior studies, the researchers created the prediction scoring systems for UTI mostly in acute ischemic stroke, but our scoring systems can be applied to the patients with all types of stroke. Previous studies reported the effects of prophylactic antibiotics use in acute ischemic stroke patients [25–27].

Unfortunately, the clinical trials used in the meta-analysis were not designed to investigate the efficacy of using prophylactic antibiotics to reduce poststroke infections. Further study is needed to evaluate it in stroke patients in preventing hospital-acquired infections and improving outcomes [26, 27]. However, according to the optimal cutoff score point developed by us, which is 3 for stroke patients, we can easily decide the indications of prophylactic antibiotics to prevent UTI in any type of stroke.

Our study has limitations of retrospective nature. The samples were derived from a single hospital. Due to the high prevalence of UTI after stroke and its impact on the outcome of stroke, we focused on developing the UTI prediction scoring system. Despite its limitations, our study is a unique UTI prediction score for all types of stroke in our country. We hope it will help the doctors who are treating stroke patients to predict the occurrence of UTI after stroke and take prompt measures to prevent it.

## Figures and Tables

**Table 1:** Baseline characteristics of patients.

Characteristics	UTI		*p* value
Type of stroke	No (*n* = 1008)	Yes (*n* = 488)	<0.001
Cerebral thrombosis, *n* (%)	304 (30.2)	96 (26.7)	
Cerebral embolism, *n* (%)	80 (7.9)	68 (13.9)	
Intracerebral hemorrhage, *n* (%)	408 (40.5)	240 (49.2)	
Subarachnoid hemorrhage, *n* (%)	216 (21.4)	84 (17.2)	
Age	56.4 ± 7.2	59.0 ± 12.8	<0.001
Gender, *n* (%)			
Female	244 (24.2)	176 (36.1)	<0.001
Male	764 (75.8)	312 (63.9)	
Season			
Spring	400 (39.7)	192 (39.3)	<0.001
Summer	176 (17.5)	96 (19.7)	
Fall	48 (4.8)	64 (13.1)	
Winter	384 (38.1)	136 (27.9)	
Smoking			
No smoking and SI<100	440 (64.7)	304 (76)	<0.001
100 ≦ SI<200	156 (22.9)	32 (8.0)	
200 ≦ SI<300	68 (10.0)	16 (4.0)	
300 ≦ SI	16 (2.4)	48 (12.0)	
Alcohol			
No	620 (61.5)	288 (59.0)	<0.001
<25 g/d	96 (9.5)	16 (3.3)	
>25 g/d	292 (29.0)	184 (37.7)	
Diabetes mellitus			
No	916 (90.9)	439 (90.0)	0.252
Yes	92 (9.1)	49 (10.0)	
Poststroke hyperglycemia			
No	903 (89.7)	325 (66.6)	<0.001
Yes	120 (10.3)	163 (33.4)	
Indwelling of urinary catheter			
No	851 (84.4)	137 (28.0)	<0.001
Yes	157 (15.6)	351 (72.0)	
GCS on admission	11.2 ± 3.9	8.5 ± 4.0	0.038
NIHSS on admission	12.8 ± 7.3	19.0 ± 9.1	0.105
WFNS (in SAH) on admission	2.9 ± 1.7	3.5 ± 1.5	<0.001

UTI: urinary tract infection, GCS: Glasgow Coma Scale, NIHSS: National Institute of Health Stroke Scale, and WFNS: World Federation of Neurosurgeons.

**Table 2:** Multivariate logistic regression analysis for probability of UTI in patients with stroke.

	Β coefficient	OR	95% CI		*p* value
			Lower	Upper	
Age ≧ 58	0.538	1.712	1.375	2.132	<0.001
Female	0.569	1.766	1.397	2.233	<0.001
GCS on admission ≦ 11	1.034	2.811	2.243	3.524	<0.001
SI ≧ 300	1.912	6.764	3.799	12.043	<0.001
Alcohol > 25 g/day	0.360	1.433	1.31	1.815	0.003
Indwelling urinary catheter	2.446	11.545	8.938	14.911	<0.001
Poststroke hyperglycemia	1.187	3.277	2.410	4.455	<0.001

**Table 3:** *β* Coefficients and points of factors.

	*β* coefficient	Points
Alcohol > 25 g/day	0.36	1
Age ≧ 58	0.538	1
Female	0.569	1
GCS on admission ≦ 11	1.034	1
Poststroke hyperglycemia	1.187	1
SI ≧ 300	1.912	1
Indwelling urinary catheter	2.446	2
Total		8

## Data Availability

No underlying data were collected or produced in this study.
